# Risk Factors Associated With Anastomotic Stricture in Patients Undergoing Minimally Invasive Esophagectomy: Experience From a High-Volume Center

**DOI:** 10.7759/cureus.66362

**Published:** 2024-08-07

**Authors:** Ali Abaid, Talha Javed, Fahad Yasin, Fatima Maqbool, Shahid Khattak, Aamir Syed

**Affiliations:** 1 Surgery, Shaukat Khanum Memorial Cancer Hospital and Research Centre, Lahore, PAK; 2 Surgical Oncology, Shaukat Khanum Memorial Cancer Hospital and Research Centre, Lahore, PAK

**Keywords:** cancer center in pakistan, mie: minimally invasive esophagectomy, esophagectomy, esophageal cancer, anastomotic stricture

## Abstract

Background: Esophageal cancer is a prevalent cancer, with a high incidence in low socioeconomic category countries. Minimally invasive esophagectomy is increasingly being used to treat this malignant condition. However, anastomotic stricture is a serious complication post esophagectomy. The study aims to enhance diagnostic consistency, improve treatment methods, guide patient management, stratify outcomes, and offer evidence-based preventive interventions.

Methods: A retrospective analysis of 550 patients who had minimally invasive esophagus surgery was carried out at Shaukat Khanum Memorial Cancer Hospital and Research Centre in Lahore between 2015 and 2020. All patients were treated with radical resection. For tumors of the lower esophagus and gastroesophageal junction, transhiatal esophagectomy was used; for tumors of the middle and upper thoracic esophagus, right video-assisted thoracoscopic surgery (VATS) was used in a three-stage procedure. Patients were routinely followed up two weeks after discharge, then every three, six, and 12 months.

Results: The mean age and BMI were 46.7 years and 19.4 kg/m^2^, respectively. Anastomotic leaks were rare, with only 13 patients experiencing them. The grade of tumor differentiation was poor, moderate, and well-differentiated. The study found that older age, high Eastern Cooperative Oncology Group (ECOG) performance status, and malignancies located in the upper and middle one-third of the esophagus had significant associations with anastomotic stricture. However, some variables, like sex, did not show significant associations in either analysis.

Conclusion: The study reveals that factors such as older patient age, high ECOG performance status, single comorbidity, and malignancies located in the upper and middle one-third of the esophagus significantly influence anastomotic stricture. The study suggests that measures against anastomotic stricture such as endoscopic procedures and minimally invasive esophagectomy should be implemented to minimize the complications.

## Introduction

Esophageal cancer, a highly malignant condition, was responsible for nearly 0.6 million new diagnoses and 0.54 million deaths worldwide in 2020 [1]. It rates as the 6th most prevalent cause of cancer-related mortality and as the eighth most often diagnosed type of cancer [2]. It is more common in low socio-economic countries, with men accounting for 70% of cases, and it is more prevalent among middle-aged and older people [3]. Esophageal cancer is increasingly being treated using minimally invasive esophagectomy (MIE) [4]. Regarding mortality, anastomotic leak rate, oncologic outcomes, and perioperative blood loss, MIE is superior to open esophagectomy [5]. Additionally, it shortens hospital stays, lessen respiratory infections, and enhance the quality of life [6]. In the last five years, the percentage of MIE has risen substantially from 26.9% to 55.9% [4]. MIE has positive effects such as less trauma, fewer hospital stays, quicker healing after surgery, easier repeatability, and tumor dissection [7].

Anastomotic stricture (AS) is a serious complication of MIE characterized by dysphagia and has an anastomotic orifice diameter of less than 5 mm on an esophagogram, with a prevalence rate ranging from 10% to 56% [8]. AS considerably influences global quality of life, negatively affecting multiple health-related quality of life (HRQoL) measures, while its impact on postoperative HRQoL is minimal [9]. The frequency of anastomotic complications has reduced significantly in recent times, with leaking being a major concern. Up to 40% of patients experience postoperative stricture, which raises the risk of death and morbidity [10]. Proper anastomotic technique is essential to minimizing complications. To reduce risk, several ways have been optimized, including hand-sewn, totally mechanical, and semi-mechanical esophagogastrostomy [11]. In MIE, patient variables and perioperative care techniques related to AS include age, sex differences, comorbidities such as diabetes, cardiovascular and pulmonary diseases, and malnourishment [12,13]. Neoadjuvant treatment, like cardiac resynchronization therapy (CRT), is often used for a large percentage of patients [14]. Different surgical procedures are used; some combine open surgery with minimally invasive methods, while others use only minimally invasive surgery [15].

The optimal technical approach for establishing anastomosis between the replacement conduit and residual esophagus, however, remains a topic of debate. Anastomotic strictures are often benign but might be related to malignancy. They usually develop following surgery. Early strictures are most prevalent after three months; however, dilatation occurs in the first year [16]. Progressive improvement is possible in symptoms, including dysphagia. It is possible to lower the frequency of benign anastomotic strictures by using proton pump inhibitors (PPIs) [17]. For patients with benign anastomotic strictures, balloon dilation is regarded as a safe and efficient therapy option, whereas bougienage is a conventional and successful technique for esophageal dilatation [18,19]. Major reductions in the morbidity associated with these strictures have been achieved with the introduction of safer and more effective dilatation procedures, combined stricturotomy and/or stenting [20,21]. For patients with persistent anastomotic esophageal strictures, metal stenting provides a higher improvement in quality of life at 12 months compared to recurrent balloon dilatation [22]. The incidence of AS recurrence following esophagectomy may be decreased by endoscopic dilatation in conjunction with topical steroid injection [23].

AS during MIE has been extensively studied; however, there are still gaps in its management and prevention. Lack of standard definitions and diagnostic standards, inadequate thorough risk factor analysis, scant long-term outcome studies, successful preventive techniques, creative management techniques, and the effects of neoadjuvant therapy are a few such gaps [24]. By filling up these gaps, this study will improve diagnostic consistency, advance treatment methods, guide long-term patient management, improve patient outcomes through risk stratification, and provide evidence-based preventive interventions.

## Materials and methods

This was a retrospective study carried out from January 2015 to December 2020 at the Department of Surgical Oncology at Shaukat Khanum Memorial Cancer Hospital and Research Centre (SKMCH&RC), Lahore, Pakistan. The study was approved for the evaluation of data by the Institutional Review Board of SKMCH&RC (approval number: EX-04-07-23-04) in accordance with the Declaration of Helsinki.

Sample

Non-probability convenient sampling technique was used. A total of 550 patients underwent MIE at SKMCH&RC during the study period and were included in the study. Of these, 98 (17.8%) had anastomotic site strictures and formed the Cases while the remaining 452 formed the Controls. Patients undergoing emergent surgery, colonic interposition, malignant strictures, or disease recurrences at the anastomosis site were excluded. Post-operative endoscopy and biopsy verified that there was no malignant recurrence in anastomosis.

Data collection and procedure

Patient age, gender, body mass index (BMI), residence, non-surgical complications, tumor characteristics, surgical details, perioperative outcomes, and follow-up outcomes were recorded in detail.

Transhiatal esophagectomy was performed for lower esophageal and gastroesophageal junction tumors. All patients were treated with radical resection. Three-stage surgery was performed using right video-assisted thoracoscopic surgery (VATS) for middle and upper thoracic esophageal cancers or when clinical indications/suspicions existed regarding superior mediastinum or neck lymph node metastasis. The standard operation usually started with the exploration and mobilization of esophageal lesions. Gastric conduit reconstruction consisted of creating a 4-5 cm wide gastric tube with multiple firings of a linear stapler along the greater curvature. After esophagectomy and lymphadenectomy, the subsequent esophagogastric anastomosis was performed. The anastomosis was constructed in an end-to-side fashion using interrupted 4-0 polypropylene or polydioxanone suture (PDS) in a single layer. The posterior wall was completed by an inverted suture, while an everted suture was used at the anterior wall. Once the anastomosis was completed, a nasojejunal tube was then inserted with its tip beyond the duodenojejunal junction. A nasogastric tube was also placed in the gastric conduit. Postoperative nutrition was by means of a nasojejunal feeding tube. Patients were routinely followed up at two weeks after discharge, then at three, six, and 12 months.

Statistical analysis

Preoperative, intraoperative, and postoperative data were collected from patients, and then the data were analyzed using IBM SPSS Statistics for Windows, Version 25.0 (Released 2017; IBM Corp., Armonk, New York, United States). Descriptive statistics such as age, BMI, gender, and other procedures were presented in a tabulated form. Chi-square and independent t-test were made along with the table of risk factors of anastomotic strictures, where a p-value below 0.05 was considered significant.

## Results

The study included 550 participants with a mean age of 46.7 years (SD = 10.4, range 17-78) and a mean BMI of 19.4 kg/m^2^ (SD = 3.8, range 11-38) (Table 1). The majority were from Punjab (n=151) and Afghanistan (n=272), with fewer from Sindh (n=10), Khyber Pakhtunkhwa (n=82), and Balochistan (n=35). The sex distribution was nearly equal with 279 males and 271 females. The mean endoscopic location was 30 (SD = 5.6, range 15-42), and the mean frequency of endoscopic dilatation was 3.2 (SD = 3.4, range 1-24). The mean length of surgery was five hours (SD = 1.2, range 0.2-9.2). In terms of ASA classification, five had severe systemic disease and 545 had mild systemic disease. ECOG performance status showed 19 asymptomatic, 81 symptomatic but fully ambulatory, and 450 symptomatic in bed less than 50% of the day. Most patients received Neo-adjuvant radiotherapy (neo-XRT) (n=481) and chemotherapy (neo-chemo) (n=527), while 69 and 23 did not, respectively. Regarding comorbidity status, 449 had none, 88 had a single comorbidity, and 13 had multiple. Nonsurgical complications included cardiac (n=1), hiatal hernia (n=1), hoarseness of voice (n=1), thromboembolic events (n=2), wound/diaphragm infection (n=3), pulmonary complications (n=4), and other infections (n=6), with 529 having no complications. Anastomotic leaks were rare, with 537 not experiencing them and 13 experiencing them. Tumor histology showed 88 adenocarcinomas and 462 squamous cell carcinomas. The pathological grade (p-grade) distribution was 264 pT0, 20 pT1, 63 pT2, 190 pT3, and 13 pT4. The N-grade distribution was 390 N0, 101 N1, 34 N2, and 25 N3. Tumor differentiation was poor (n=75), moderate (n=407), and well-differentiated (n=68). Suture usage was nearly equally divided between PDS (n=268) and prolene (n=282). The study population consisted of 98 cases and 452 controls (Table 1).

**Table 1:** Descriptive statistics (N=550) BMI: body mass index; ASA: American Society of Anesthesiologists; ECOG: Eastern Cooperative Oncology Group: XRT: radiotherapy; PDS: polydioxanone suture

Variables	Categories	Values
Age (years)	Mean ± SD (Min-Max)	46.7±10.4 (17-78)
BMI (kg/m^2^)	Mean ± SD (Min-Max)	19.4±3.8 (11-38)
Area of Residence	Punjab	151
	Sindh	10
	Khyber Pakhtunkhwa	82
	Balochistan	35
	Afghanistan	272
Sex	Male	279
	Female	271
Endoscopic location	Mean ± SD (Min-Max)	30±5.6 (15-42)
Frequency of endoscopic dilatation	Mean ± SD (Min-Max	3.2±3.4 (1-24)
Length of surgery	Mean ± SD (Min-Max	5 ±1.2 (0.20-9.2)
ASA	Severe systemic disease	5
	Mild systemic disease	545
ECOG performance status	Asymptomatic	19
	Symptomatic, in bed < 50% of day	81
	Symptomatic, full ambulatory	450
Neo XRT	No	69
	Yes	481
Neo chemotherapy	No	23
	Yes	527
Comorbidity status	None	449
	Single	88
	Multiple	13
Nonsurgical complication	Cardiac	1
	Hiatal hernia	1
	Hoarseness of voice	1
	Thromboembolic	2
	Wound/Diaphragm	3
	Infection	3
	Pulmonary	4
	Infection	6
	None	529
Anastomotic leak	No	537
	Yes	13
Tumor histology	Adenocarcinoma	88
	Squamous cell carcinoma	462
p-grade	pT0	264
	pT1	20
	pT2	63
	pT3	190
	pT4	13
N-grade	N0	390
	N1	101
	N2	34
	N3	25
Grade of tumor	Poor Diff	75
	Moderate Diff	407
	Well Diff	68
Suture used	PDS	268
	Prolene	282
Group	Case	98
	Control	452

With regard to comparison between cases and controls, for demographic distribution, most participants were from Afghanistan, with a notable presence from Punjab and other regions, showing no significant difference (p=0.24). Gender distribution was nearly equal in both groups, with no significant difference (p=0.345). ASA classification indicates a significant difference, with severe systemic disease more prevalent in the case group (p=0.04). ECOG performance status shows a significant difference, with more symptomatic patients in the case group (p=0.00). Neo-XRT and neo-chemo usage did not differ significantly between groups (p=0.334 and p=0.95, respectively). Comorbidity status indicated a non-significant trend towards more comorbidities in the control group (p=0.09). Nonsurgical complications showed a significant difference, particularly in pulmonary complications and overall incidence (p=0.013). There was no significant difference in the incidence of anastomotic leaks (p=0.33) or tumor histology types (p=0.487). Pathological grade (p-grade) distribution did not significantly differ (p=0.181), but N-grade showed a significant difference, with more advanced nodal involvement in the control group (p=0.037). The grade of tumor differentiation was similar between groups (p=0.290). Lastly, the type of suture used in surgeries showed no significant difference (p=0.54) (Table 2).

**Table 2:** Bifurcation of demographic and clinical characteristics with respect to case and control ASA: American Society of Anesthesiologists; ECOG: Eastern Cooperative Oncology Group: XRT: radiotherapy; PDS: polydioxanone suture

Variables	Group			P-value
	Controls	Cases	Total	
Demographic				0.24
Punjab	122	29	151	
Sindh	10	0	10	
Khyber Pakhtunkhwa	61	21	82	
Balochistan	25	10	35	
Afghanistan	234	38	272	
Sex				0.345
Male	227	52	279	
Female	225	46	271	
ASA Status				0.04
Severe systemic disease	1	4	5	
Mild systemic disease	451	94	545	
ECOG performance status				0.00
Asymptomatic	11	8	19	
Symptomatic, full ambulatory	390	60	450	
Symptomatic, in bed < 50% of day	51	30	81	
Neo XRT				0.334
No	55	14	69	
Yes	397	84	481	
Neo chemo				0.95
No	16	7	23	
Yes	436	91	527	
Comorbidity status				0.09
None	360	89	449	
Single	72	4	6	
Multiple	20	5	25	
Nonsurgical complication				0.013
Cardiac	1	0	1	
Hiatal hernia	1	0	1	
Hoarseness of voice	0	1	1	
Thromboembolic	2	0	2	
Wound/Diaphragm	3	0	3	
Infection	4	2	6	
Pulmonary	3	1	4	
Inflammation	0	3	3	
None	438	91	529	
Anastomotic leak				0.33
No	440	97	537	
Yes	12	1	13	
Tumor histology				0.487
Adenocarcinoma	73	15	88	
Squamous cell carcinoma	379	83	462	
p-grade				0.181
pT0	221	43	264	
pT1	15	5	20	
pT2	55	8	63	
pT3	150	40	190	
pT4	11	2	13	
N-grade				0.037
N0	327	63	390	
N1	77	24	101	
N2	31	3	34	
N3	17	8	25	
Grade of tumor				0.290
Poorly Differentiated	57	18	75	
Moderately Differentiated	337	70	407	
Well Differentiated	58	10	68	
Suture used				0.54
PDS	223	45	268	
Prolene	229	53	282	

In the univariable analysis, age has an odds ratio of 1.01, suggesting a very weak association with AS (p-value = 0.08). In the multivariable analysis, the odds ratio is 1.03, which is slightly higher and statistically significant (p-value = 0.05). This indicates that older age may be a risk factor for anastomotic stricture. In the univariable analysis, BMI has an odds ratio of 0.94, indicating a weak negative association (p-value = 0.05). However, in the multivariable analysis, the odds ratio is 0.95, with a slightly higher p-value (0.07). This suggests that BMI may have a weak association with anastomotic stricture, but it's not statistically significant. This variable has different categories, with "Symptomatic, in bed <50% of the day" showing a very high odds ratio in the multivariable analysis (126.4) and a highly significant p-value (0.001). This indicates a strong association with anastomotic stricture. The presence of "Single" comorbidity has a significant association with AS in both univariable and multivariable analyses. "Multiple" comorbidities do not show a significant association. The variable "Endoscopic location" has significant associations in both univariable and multivariable analyses. In the multivariable analysis, the odds ratio is 0.65, suggesting a strong protective effect and the p-value is highly significant (0.001) (Table 3). It's important to note that some variables, like "Sex," do not show significant associations in either analysis (Table 3).

**Table 3:** Risk factors of anastomotic stricture "Ref" stands for "reference category." BMI: body mass index; ASA: American Society of Anesthesiologists; ECOG: Eastern Cooperative Oncology Group: XRT: radiotherapy; PDS: polydioxanone suture

Variables	Categories	Univariable analysis, odds ratio (95% CI), p-value	Multivariable analysis, odds ratio (95% CI), p-value
Age	Mean ± SD	1.01 (0.99-1.04), 0.08	1.03 (1.0-1.1), 0.05
BMI	Mean ± SD	0.94 (0.87-1.01), 0.05	0.95 (0.90-1.03), 0.07
Sex	Female	Ref	Ref
	Male	1.12 (0.72-1.73), 0.61	-
ASA	Mild (II)	Ref	Ref
	Sever (III)	19.2 (2.1-173-6), 0.009	-
ECOG performance status	Asymptomatic	Ref	Ref
	Symptomatic, full ambulatory	0.21 (0.08-0.55), 0.001	0.18 (0.04-0.81), 0.02
	Symptomatic, in bed <50% of day	0.81 (0.30-2.23), 0.68	126.4 (17.5-910-12), 0.001
Neo XRT	No	Ref	Ref
	Yes	0.83 (0.44-1.6), 0.56	-
Neo chemo	No	Ref	Ref
	Yes	0.47 (0.19-1.20), 0.11	-
Comorbidity	None	Ref	Ref
	Single	0.22 (0.08-0.63), 0.005	0.20 (0.001-0.1), 0.001
	Multiple	1.01 (0.37-2.76), 0.98	1.10 (0.22-4.91), 0.09
Length of surgery	Mean ± SD	0.89 (0.75-1.10), 0.22	Ref
Nonsurgical complications	Absent	Ref	Ref
	Present	2.41 (0.94-6.13), 0.06	-
pT grade	pT0	Ref	Ref
	Early (pT1 and pT2)	0.95 (0.48-1.87), 0.89	-
	Advance (pT3 and pT4)	1.34 (0.84-2.15), 0.22	Ref
N-stage	No involvement	Ref	ref
	Involvement present	1.45 (0.92-2.31), 0.11	-
Grade	Well-differentiated	Ref	Ref
	Poorly differentiated	1.82 (0.78-4.31), 0.16	-
	Moderately differentiated	1.20 (0.56-2.50), 0.61	-
Suture used	PDS	Ref	Ref
	Prolene	1.15 (0.74-1.78), 0.54	-
Endoscopic location	Mean ± SD	0.78 (0.74-0.82, 0.001)	0.65 (0.60-0.72), 0.001

## Discussion

This study had two main objectives, i.e., determining the different risk factors associated with AS and management of AS at a high-volume center. When the risk factors for AS were identified, it was concluded that in the univariable analysis, age (OR=1.01, p=0.08) and BMI (OR=0.94, p=0.05) showed weak associations with anastomotic stricture. Multivariable analysis indicated that older age (OR=1.03, p=0.05) and ECOG "Symptomatic, in bed, <50% of the day" (OR=126.4, p=0.001) were significant risk factors. Single comorbidity was a significant protective factor (OR=0.20, p=0.001). ASA, nonsurgical complications, and advanced pT grade were significant in the univariable analysis but not included in the multivariable model. Endoscopic location showed a strong protective effect in both analyses (multivariable OR=0.65, p=0.001). Variables like sex, neo-XRT, neo-chemo, length of surgery, N-stage, tumor grade, and suture type were not significant.

The results are supported by various studies including one by Simitian et al., in which the authors noted the presence of anastomotic leak in 20.3% of their patients [25]. They did not find a predictable relationship between anastomotic leak and patient characteristics, prior comorbidities, and the neoplasm type (histological type, size, and TNM (tumor, node, metastasis) staging). This was 3% higher when compared to non-diabetic patients although it was not statistically significant (p = 0.084). Furthermore, patients with insulin-dependent diabetes preoperatively had an overall high rate of anastomotic leak of 33%. However, considering the above critique, it is possible to agree that the current review of the literature was successful in revealing several potentially significant links between operative variables and anastomotic leak development [25].

To be specific, the anastomotic leakage rate was 18.5% in patients who underwent neoadjuvant chemoradiotherapy, 30.8% in those who did not receive any neoadjuvant treatment, and 50.7% in patients who received only neoadjuvant chemotherapy without radiotherapy [26-28]. However, the overall negative or no association found in this study is inconsistent with the multitude of studies that either do not reveal any difference or suggest a positive correlation with anastomotic leak following neoadjuvant treatment. However, in a recent study by Na et al., end anastomosis stricture was diagnosed in one-fourth of the patients who underwent esophagectomy [29]. The patient population for this study consisted of 737 patients who received esophagectomy for esophageal carcinoma requiring stomach conduits. Four types of anastomoses were used: manual sewing (n = 221, 30%), circular stapling (n = 172,23%), hybrid linear stapling (HLS) (n = 155, 21%), and triangular linear stapling (TLS) (n = 189, 26%). Logistic regression analysis was done to determine the risk factors that were associated with stricture, using different variables. Endoscopic dilatations were done within one year of operation in at least 105 patients (14%), and at least 13% of the strictures seen were due to leakage. The multivariate analysis's hazard ratio (HR) analysis also revealed that factors other than TLS (manual sewing: preoperative and postoperative hazard ratio) and circular stapling were found to be significant (P < 0.00) risks for stricture development, with OR 9.58 (95%CI 4.95-23.42), OR 6.51 (95%CI 3.83-20.99), and OR 5.462 (95%CI 3.07-15.52), respectively. These factors were also found to have contributed significantly to increased mortality among patients with congestive heart failure. TLS significantly reduced the stricture rate (3.2%) compared with other techniques (manual sewing: 19.81±8.19%; circular stapling: 13.23±7.14%; HLS: 15.95±7.57%; P < 0.001). The stricture rate differed significantly in the TLS group (2.19%) in patients without leakage and 6.03% in the control group (P < 0.001). However, with leakage, it became 16.32% in the TLS group and 8.54% in the control group. Patient factors included COPD, leakage, and anastomosis technique as contributors to stricture. A larger anastomosis area, which is achievable with the TLS technique that requires 60 mm length linear staplers, has been associated with a lower rate of anastomotic stricture formation, provided no leakage is observed [29].

The stricture management results are supported well by the study by Vetter and Gutschow [30], which reported that the value of the leakage rate in a large retrospective multicenter study including 966 patients, who underwent thoracoscopic-laparoscopic MIE was significantly higher after intrathoracic end-to-side double-stapling (23.3%) and cervical end-to-side hand-sewn (25.1%) compared with intrathoracic side-to-side linear (15.6%), purse-string (13.9%). Analyzing multiple variables, again, the anastomotic technique was proven to be a leakage-independent predictor [30]. Furthermore, there is the development of endoluminal vacuum therapy (EVT) which is preferred in many developed hospitals. In EVT, a polyurethane sponge connected to the hose is introduced to the anastomotic area through an endoscopic method. In postpartum cases, after vacuum application, the sponge removes the leakage cavity as well as the secretions and necrosis and thus gives 80-90% wound healing [31,32]. 

This study may also have an inherent bias, including selection bias, due to the retrospective design of the study, as data was collected from existing records, which again may not represent the general patient population. The investigation has been carried out at a single center, which restricts the extrapolation of the results to other centers that may have different patient mix and surgical profiles. Also, this may account for disparities in perioperative management and technical differences in conducting the surgery which might lead to divergent results that cannot be easily compared to other facilities. Therefore, large-scale multi-center prospective studies should be carried out in the future to confirm these observations along with an additional detailed understanding of the anatomical features, which are instrumental to AS identification and management.

## Conclusions

Various factors are seen to significantly influence AS, where older patient age and ECOG performance statuses are predictor indicators, and single comorbidity and specific endoscopic locations are preventive indicators. The other covariates were sex, neo-XRT, neo-chemo, length of surgery, N-stage, tumor grade, and the suture material used, which were not statistically significant. The evaluation of the various aspects underlying the formation of AS shows their multifaceted and heterogeneous nature. Measures against AS like endoscopic procedures and MIE should be put in place to ensure minimal impact of the complication on the patients.
